# 

**Published:** 1887-08-06

**Authors:** 


### CONTENTS.

**"A Hospital Scandal."** - - - - - 307

**Words of Consolation.**—XLIV. The Sufferings of Christ - - - - - - - 308

**Hospitals for Children** - - - - 309

**Fever Hospitals in Large Towns** - - - 310

**The Doctor's Pulpit**: A Children's Sermon.- Fairy Rings and Ringworms - - - - - - 311

**The National Pension Fund** - - - - 312

**Chats About Medical Museums.** By One of the Crowd.—VIII. The London Hospital - - - 313

**Notes and News.** — Miscellaneous Jottings. — The Royal Isle of Wight Infirmary.—The Beau Site Convalescent Home. — Bazaars. — Vacancies. — Church Parades. — The New Hospital at Blackpool. — The Medical Naval Service.—The Swindon Dissension.—The Tharia Topun Hospital. — A Garden Party.— Hospital Reports. — Friendly and Trade Societies' Demonstrations.—Trained Nurses and the Royal Berks Hospital, etc., etc. - - - - - - - 314

**Annotations.**—Medical Men and Infectious Diseases. —Infectious Hospitals.—To Avoid Infection - - 317

**Ethel's Experiment.**—By E. A. Brierly - - 318

**The Condition of Midwives.** By Mrs. E. A. Bedingfeld - - - - - - - 321

**Incidents of Animal Life.**—A Parrot Story.— A Dog as an Out-Patient - - - - - - 322

**Questions and Answers.**—May a Young Lady be detained in an Asylum against her Parents' Wishes ? - 322

**Till the Doctor Comes.**—XXIII. Treatment of Emergencies - - - - - - - - 323

**The Children's Corner.**—Puzzles, Answers, etc. - 324

**Amusements with Profit** - - - - - 324

